# Replication Compartments—The Great Survival Strategy for Epstein–Barr Virus Lytic Replication

**DOI:** 10.3390/microorganisms10050896

**Published:** 2022-04-25

**Authors:** Atsuko Sugimoto

**Affiliations:** 1 Department of Infectious Diseases and Immunology, Clinical Research Center, National Hospital Organization Nagoya Medical Center, Nagoya 460-0001, Japan; atsuko.sugimoto@nnh.go.jp; 2 Department of Virology and Parasitology, School of Medicine, Fujita Health University, Toyoake 470-1192, Japan

**Keywords:** Epstein–Barr virus, lytic replication, replication compartment, replication, transcription, DNA damage response, viral pre-initiation complex (vPIC)

## Abstract

During Epstein–Barr virus (EBV) lytic replication, viral DNA synthesis is carried out in viral replication factories called replication compartments (RCs), which are located at discrete sites in the nucleus. Viral proteins constituting the viral replication machinery are accumulated in the RCs to amplify viral genomes. Newly synthesized viral DNA is stored in a subdomain of the RC termed the BMRF1-core, matured by host factors, and finally packed into assembled viral capsids. Late (L) genes are transcribed from DNA stored in the BMRF1-core through a process that is mainly dependent on the viral pre-initiation complex (vPIC). RC formation is a well-regulated system and strongly advantageous for EBV survival because of the following aspects: (1) RCs enable the spatial separation of newly synthesized viral DNA from the cellular chromosome for protection and maturation of viral DNA; (2) EBV-coded proteins and their interaction partners are recruited to RCs, which enhances the interactions among viral proteins, cellular proteins, and viral DNA; (3) the formation of RCs benefits continuous replication, leading to L gene transcription; and (4) DNA storage and maturation leads to efficient progeny viral production. Here, we review the state of knowledge of this important viral structure and discuss its roles in EBV survival.

## 1. Introduction

Epstein–Barr virus (EBV) is a Gammaherpesvirinae family member and a human lymphotropic herpesvirus. EBV is widely spread among humans and transmitted via saliva, mainly during childhood. EBV is associated with various tumors, such as Hodgkin’s lymphoma, diffuse large B-cell lymphoma, Burkitt lymphoma, and NK/T-cell lymphoma [1,2,3,4,5]. Furthermore, it is closely linked to gastric cancer, nasopharyngeal carcinoma, and breast cancer, as it can also infect epithelial cells [6,7,8]. Epidemiologically, human tumors associated with EBV are now estimated at around 200,000 cases per year [1]. Upon primary infection, EBV infects resting B lymphocytes to establish a lifelong continuous latent infection without the production of progeny viruses, while expressing a limited set of genes (latent genes) [9,10,11]. On the other hand, productive replication is initiated by the expression of BZLF1, an EBV-coding lytic switch gene that induces reactivation [12,13]. Lytic reactivation occurs spontaneously and requires the EBV origin of lytic replication (oriLyt) that lies on the viral genome [14,15]. During lytic replication, large numbers of lytic genes are sequentially expressed. Depending on the stage of lytic replication, these genes are classified into immediate early (IE), early (E), and late (L) genes. The EBV genome is amplified 100- to 1000-fold by rolling circle replication using the EBV replication machinery. This machinery consists of EBV-coding replication proteins, which are E gene products [16]. The synthesized viral DNA is produced as DNA concatemers consisting of multiple head-to-tail repeats of single EBV genome units [17]. This concatemeric DNA is cleaved, and a single unit of viral DNA is packed into assembled capsids using capsid packaging proteins, thus producing viral particles [18].

The major feature of EBV lytic replication is the formation of a replication compartment (RC) [19,20,21]. RCs are viral replication factories composed of viral replication proteins that appear at discrete sites in the nuclei during lytic replication [22]. Since viral genomes are synthesized in RCs, RCs enlarge and appear as large globular nuclear subdomains at the late stage of the lytic phase [19]. In addition to DNA synthesis, RCs are sites of viral gene transcription, capsid assembly, and other regulations required to carry out lytic replication. In the RC, a sub-domain called the BMRF1-core assembles and stores the viral DNA [23]. Inhibition of RC formation by drugs such as phosphonoacetic acid (PAA) prevents the production of progeny viruses [19]. In this review, we focus on RCs and their sub-domain the BMRF1-core during the lytic phase and discuss this highly regulated system and the strong advantages of RC formation as a strategy for EBV production, including efficient genome replication, maintenance of genome stability by modification of host factors, and regulation of viral gene transcription.

## 2. RC Characterization

Various microscopic analyses have revealed that most viruses, including DNA and RNA viruses, amplify their own genomes at the site of replication [24,25,26,27]. In particular, double-stranded (ds) DNA viruses, such as *Herpesviridae*, *Adenoviridae*, *Polyomaviridae*, and *Papillomviridae*, replicate within RCs at discrete sites in the nuclei (also known as “replication centers” or “replication foci”), which are not covered by the cellular membrane. RCs may form through liquid–liquid phase separation (LLPS), like other membraneless nuclear bodies [28,29,30]. Once viral productive replication is induced, the size of RCs increases over time because viral and host replication proteins are recruited, and newly synthesized viral genomes are stored. Some inhibitors of viral replication, such as PAA, prevent RC formation [19,31]. In most cases, RCs are also the sites of viral capsid or virion assembly. This allows immediate packaging of the synthesized and stored genomes into capsids or virions [32]. Such efficient production of virions leads to the infection of more cells, thereby elevating tumorigenic potential [33]. Hence, RCs are thought to be the center of dsDNA viral productive replication and have been well-studied as therapeutic targets of infectious diseases and tumors caused by these dsDNA viruses [28,33].

In particular, the RCs of human herpesviruses have been extensively studied. Herpesviruses are widespread, adapted to human life, and are associated with various diseases and tumors. RCs are closely linked to the unique life cycles and pathogenesis of these viruses [34,35]. The life cycle of herpesviruses has two types of phases: a latent phase and a lytic phase. EBV is a Gammaherpesvirinae family member strongly associated with various human cancers [36,37]. EBV establishes lifelong latency after primary infection and immortalizes infected cells. During the latent phase, EBV genomes form episomal structures expressing a limited set of viral genes, called latent genes, in the absence of productive replication or RC formation. The switch from latency to lytic replication (termed “reactivation”) occurs spontaneously, starting with the expression of BZLF1, an EBV IE gene. During the lytic phase, herpesviruses compose their own replication machinery, the RCs, which mainly consist of herpesvirus replication proteins [22]. In the case of EBV, BMRF1 (DNA polymerase processivity factor and dsDNA binding protein), BALF2 (single-stranded (ss) DNA binding protein), BALF5 (DNA polymerase), and BBLF4/BSLF1/BBLF2/3 (the helicase–primase complex) have been identified as replication proteins and are known as the main components of RCs. These proteins are well-conserved among human herpesviruses [22]. Although BZLF1 acts as a lytic switch mediator at the early stage of the lytic phase and is distributed diffusely throughout the nuclei, BZLF1 moves to the RCs to associate with viral DNA and BBLF4 [21,34]. In addition to replication proteins and lytic regulators, viral factors, such as BKRF3 (uracil DNA glycosylase), BGLF4 (protein kinase), and BPLF1 (deubiquitinase), also accumulate in RCs [38,39,40] (listed in Table 1). BKRF3 is required for viral DNA synthesis, along with BALF5 and BMRF1 interaction [38]. The advantages of BGLF4 and BPLF1 accumulation to RCs are meticulously described in Section 3 and Section 5. EBV infects both B lymphocytes and epithelial cells, and RCs can be observed in both types of cell lines upon artificial lytic induction. Similar to what happens with other dsDNA viruses, host factors are also recruited to RCs to participate in viral replication, transcription, or capsid assembly; their details will be described later.

## 3. RC Growth and Maintenance

**Figure 1:**
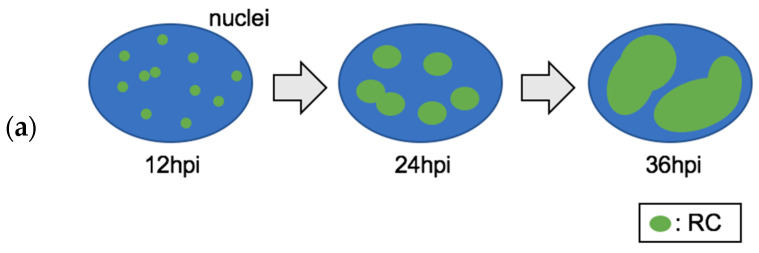

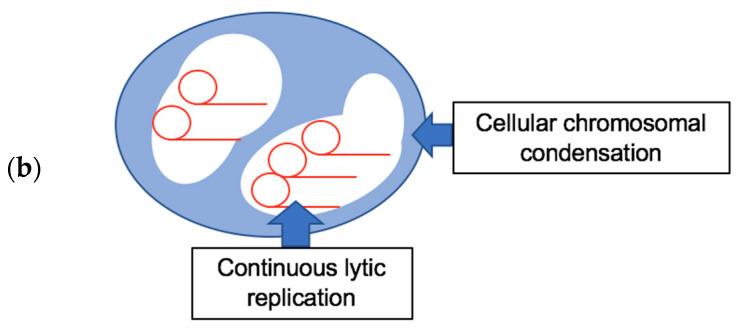
(**a**) The formation and growth of RCs over time. These small RCs, seem at 12 hpi, grow bigger and seem to fuse with each other as lytic replication proceeds (24 hpi). At 36 hpi, RCs appear as one or two large globular nuclear subdomains. (**b**) Model showing the development of RCs and their occupation of extrachromosomal space.

Fluorescence in situ hybridization (FISH) analysis using EBV latently infected cell lines has visualized the EBV genome, which appears as an enormous number of small dots. Until EBV establishes latency from primary infection, EBV amplifies its genome, and each single genome forms a circular episome that binds to AT-rich and gene-poor regions of human chromosomes via EBV nuclear antigen (EBNA1), a latent EBV gene [48,49]. EBNA1 bridges the EBV episome and cellular chromosomes, binding EBV to the latent origin of replication (ori-P). On the other hand, in the early phase of lytic replication, several medium-sized dots, so to say primary RCs, can be observed by FISH analysis or immunofluorescence analysis (IFA) using specific antibodies for viral replication proteins (Figure 1a, 12 h post induction (hpi)). These small RCs grow bigger and seem to fuse with each other as lytic replication proceeds (Figure 1a, 24 hpi). Finally, at the late stage of lytic replication, RCs appear as one or two large globular nuclear subdomains and occupy 30–35% of the nucleus (Figure 1a, 36 hpi) [50]. As mentioned before, RCs are generally characterised by FISH analysis using probes specific to EBV genomes or by IFA using specific antibodies for BMRF1, BALF5, or BALF2 with fixed cells [19,23]. These methods cannot monitor RC growth in a single cell over time. The question is, however, how individual episomes grow into such an enormous nuclear subdomain. Sugden’s group had developed the visible replicon system, which encodes the EBV ori-P, oriLyt, and LacI-LacO targeting system fused tdTomato fluorescence protein containing a nuclear localization signal to monitor viral DNA synthesis by time-lapse imaging [51,52]. Nagaraju et al. explored the question by using this EBV replicon system to visualize the development of RCs in live cells. Surprisingly, each developing RC contains similar levels of viral DNA during the same stage of lytic replication. This suggests that each developing RC synchronises with other RCs. Moreover, the nuclear volumes are enlarged at the late stage of lytic replication [50]. It has also been observed that 4′,6-diamidino-2-phenylindole (DAPI) does not stain the sites of RCs, and electron microscopic analysis has shown that host chromatin is shoved by RCs and condensed [42]. Thus, for RC development and successful viral production, the extrachromosomal space is essential (Figure 1b). BGLF4 EBV-coded protein kinase, which is expressed in the early stage of lytic replication and is mostly located in RCs, interacts with the condensin complex and stimulates topoisomerase II, which conducts chromosomal condensation similar to a premature mitotic event and provides extra space for RCs [53]. BGLF4 kinase inactivates MCM4-MCM6-MCM7 helicase activity by phosphorylating MCM4, which blocks host chromosomal DNA replication and allows RCs to occupy more space [54]. Even in the late stage of lytic replication, DNA synthesis occurs continuously, maintaining the morphology of the RCs [55]. Some BZLF1-inducible host factors, such as target-of-rapamycin complex 2 (TORC2), localize to RCs [56]. These factors may contribute to the continuous DNA replication in RCs. EBNA1, an EBV latent protein, is also expressed during the lytic phase and accumulates in RCs. EBNA1 is thought to bind to the ori-P region on newly synthesized DNA stored in RCs, which functions as a scaffold to maintain the RC structure in the nuclei [57]. Interestingly, even the exogenous gene coding ori-Lyt is amplified in RCs coordinated with RCs [58]. This evidence allows us to imagine RCs, which successfully gain enough space and separate the nucleus spatiotemporally, benefiting the protection of both newly synthesized EBV genomes and assembled viral capsids and the coordination among single episomes.

## 4. The DNA Storage Subdomain, the BMRF1-Core

**Figure 2:**
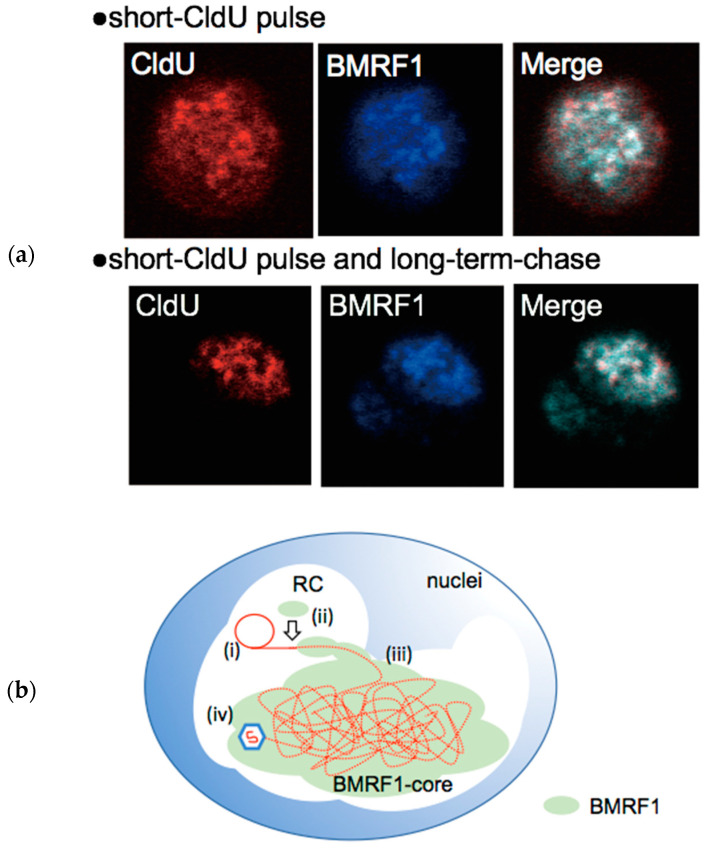
(**a**) Pulse-chase analysis revealing the existence of the RC subdomain, the BMRF1-core. The lytic phase was induced in Tet-Z/B95.8 cells by Dox treatment. At 24 h post-induction, the cells were treated with CldU for 10 min (short-CldU pulse). Then, cells were washed and incubated for 1 h to allow labelled DNA to move to DNA storage sites (short-CldU pulse and long-term chase). Approximately 40 cells were analyzed and images were captured. (**b**) Model of BMRF1-core formation. (i) The newly synthesized viral DNA mainly at the outside of the BMRF1-core in the RC. (ii) BMRF1 binds to synthesized viral DNA and folds to form the BMRF1-core that seems to be accumulated in the BMRF1-core. (iii) Viral DNA is stored until packed into the viral capsid. (iv) Finally, viral DNA is packed into the self-assembled capsid.

As mentioned before, viral replication proteins are recruited to the RC and compose replication forks in the RCs. BMRF1 is a multifunctional EBV replication protein that plays a central role in lytic replication via RCs. It acts as a DNA polymerase processivity factor, a dsDNA-binding protein, and a lytic gene transcription factor. BMRF1 is considered an E gene and is highly expressed through the lytic phase. Electrophoresis, sedimentation assay, and blue native PAGE analysis have revealed that BMRF1 mostly exists as a C-shaped head-to-head homodimer connecting the βI1 strands and requires C95. Such dimeric forms possess dsDNA binding activity [59]. However, some BMRF1 molecules form a ring-shaped tetramer via tail-to-tail contact, which contributes to viral replication [60]. Mutations lacking the dsDNA binding capacity fail to form RCs. Therefore, in RCs, it is suspected that newly synthesized viral dsDNA is covered by BMRF1. We previously discovered that BMRF1 binding to dsDNA occurs at discrete areas of the RCs, and, using confocal microscopic analysis and 3D reconstruction, found that BMRF1 seems to be surrounded by other viral replication proteins such as BALF2 or BALF5 [23]. This evidence suggests that there are BMRF subdomains in RCs, which we named the “BMRF1-core”. The BMRF-core has been observed at 24 h post-lytic induction when using the B95.8 cell line. Viral DNA labelling experiments using thymidine analogues have revealed short- 5-chloro-2’-deoxyuridine (CldU) pulse-labeled viral DNA outside the BMRF1-core, where BALF2 or BALF5 are also located (Figure 2a, upper panels). In contrast, short-CldU pulse and long-term-chase-treated viral DNA move to the inside of the BMRF1-core (Figure 2a, lower panels). Hence, these observations suggest that during lytic replication, newly synthesized viral DNA is bound by BMRF1, which possesses dsDNA binding capacity, folded to form the BMRF1-core, and stored until packed into the viral capsid (Figure 2b). In fact, our previous study indicated that the BMRF1-core is the site of capsid assembly. We observed that EBV capsid packaging proteins are localized inside the BMRF1-core, although EBV capsid proteins are located outside and inside. These results suggest that EBV viral empty capsids are assembled in RCs and transported to the BMRF1-core, and that the packed BMRF1-core stores viral DNA into the capsids [42]. The BMRF1-core divides the area of the RCs spatiotemporally, which allows for efficient capsid assembly.

## 5. The Contribution of DNA Damage Responses to Viral DNA at RCs

**Figure 3:**
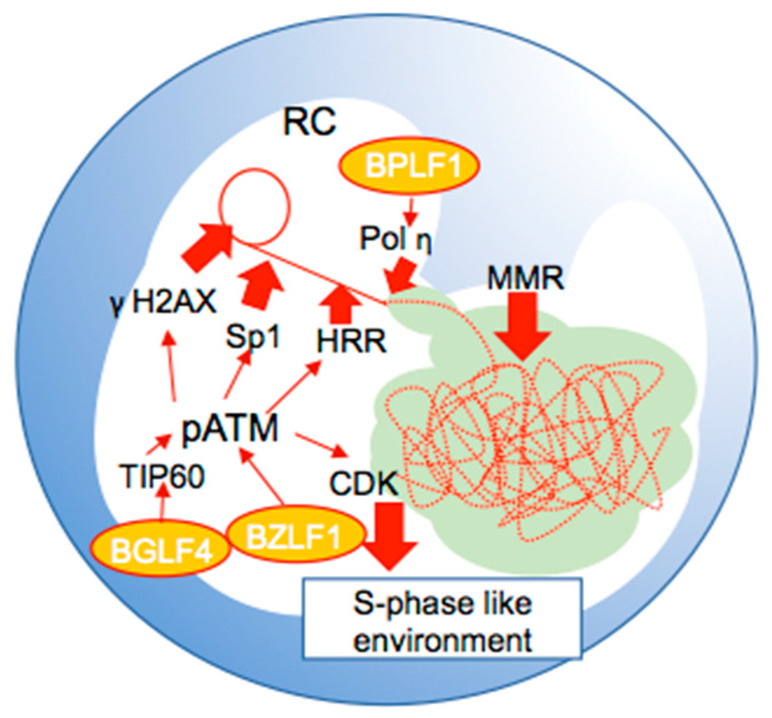
Model of the interactions among DNA damage response factors which enhance EBV DNA synthesis and viral proteins at RCs. The central kinase of the DDR, ATM, is activated by BGLF4 and BZLF1. Phosphorylated ATM (pATM) activates H2AX (γH2AX), S-phase cyclin-dependent kinases (CDKs), Sp1, and homologous recombinational repair (HRR) enhance viral DNA synthesis. BPLF1 recruits pol η onto newly synthesized viral DNA to mature. Mismatch repair (MMR) factors are also involved in the maturation of viral DNA stored at the BMRF1-core.

Genomes packed into capsids should maintain high fidelity to ensure EBV survival. Herpesviruses hijack and/or utilize the host DNA damage response (DDR) and DNA repair system to assist their replication (Figure 3). Some lytic genes are DDR inducers, and their main target is ataxia telangiectasia-mutated (ATM), the central kinase of the DDR. ATM normally exists as a homodimer or as a multimer inactive form. Once DNA damage is sensed, ATM rapidly autophosphorylates at serine 1981, which causes dimer dissociation [61]. The activation of ATM by autophosphorylation initiates ATM kinase activity. BGLF4 phosphorylates and activates TIP60 histone acetyltransferase, promoting DDR through the acetylation of ATM, which causes the phosphorylation of ATM, H2AX, and downstream factors [62]. BZLF also induces phosphorylation of ATM, H2AX, and 53BP1 independently of other lytic proteins [63]. BGLF4- and BZLF1-DDR induction activities are modulated by SUMO binding [64]. Most activated DDR factors, such as phosphorylated ATM (pATM) and phosphorylated H2AX (γH2AX), are recruited to RCs upon lytic infection [63,65,66]. S-phase cyclin-dependent kinases (CDKs) are highly activated via ATM checkpoint signaling, one of the downstream pathways of ATM that creates an S-phase-like intracellular environment [65]. The phosphorylation of Sp1, a host transcriptional factor, by ATM is also important for viral DNA synthesis and RC maintenance [66]. ATM also facilitates DNA repair in a manner similar to that of homologous recombination repair (HHR). As newly replicated viral DNA possesses double-strand breaks (DSBs), the DSB sensor activated by ATM (the Mre11–Rad50–Nbs1 (MRN) complex) binds to the DSB region. The MRN complex promotes sequential HHR to repair DSBs in newly synthesized viral DNA, cooperating with viral lytic replication [67]. Proliferating cell nuclear antigen (PCNA), a DNA sliding clamp, is loaded onto newly synthesized viral DNA, and a series of mismatch repair (MMR) factors are recruited to viral DNA in RCs [68]. Factors belonging to the MMR pathway have been observed inside the BMRF1-core, although HHR factors are located both outside and inside the BMRF1-core [23]. This suggests that MMR is involved in the maturation of the newly synthesized viral DNA. The BMRF1-core plays a part in spatiotemporally dividing the different pathways. Studies on the accumulation of such DDR factors to herpesviral RCs have been conducted in other herpesviruses, such as herpes simplex virus type 1 (HSV-1) and human cytomegalovirus (HCMV). RCs of HSV-1 or HCMV are also formed; HRR factors, including the MRN complex and Rad51, are recruited to the RCs [69,70,71,72,73,74]. In addition, BPLF1, an EBV-coded deubiquitinase, recruits polymerase eta (pol η), which is the host polymerase that specializes in DNA repair, onto newly synthesized viral DNA during the lytic phase to mature but not synthesize viral DNA [75]. Furthermore, BPLF1 enhances pol η expression, likely by inhibiting proteasomal degradation through its deubiquitinase activity.

On the other hand, EBV lytic replication triggers and utilizes the DDR for genome integrity and fidelity. Recent studies have shown that EBV also interferes with some part of the DDR to avoid apoptosis. BZLF1 causes mislocalization of 53BP1 and RNF8, the latter being a ubiquitin–protease ligase that acts in the DNA repair pathway, but not those of pATM, MDC1, or γH2AX from DSBs [76]. BMRF1 interacts with the nucleosome remodeling and deacetylation (NuRD) complex at RCs, which prevents the accumulation of the essential RING finger ubiquitin ligase for DNA repair, RNF168, in sites of DBSs, mainly in the host genome [77]. Additionally, the BKRF4 EBV tegument protein, which is expressed at the late stage of the lytic phase and is not located in RCs, interferes with histone ubiquitination at DSBs by inhibiting the recruitment of RNF168 [78,79]. Likewise, DDR factors, such as γH2AX, Mre11, and RPA32, are recruited to Kaposi’s sarcoma-associated herpesvirus (KSHV) RCs and not 53BP1 [80]. Taken together, these phenomena may contribute to the herpesvirus-specialized forms of DDR and DNA repair, and indirectly protect newly synthesized viral DNA stored in RCs. Mislocalization of DNA repair molecules from DSBs in host DNA induces cellular genome instability [81,82,83]. The formation of RCs and the specialization of a set of DDR factors at RCs indirectly contribute to EBV-driven oncogenesis by promoting viral genome stability and host genomic instability, even during the lytic phase [33].

## 6. The Regulation of L Gene Transcription in RCs

**Figure 4:**
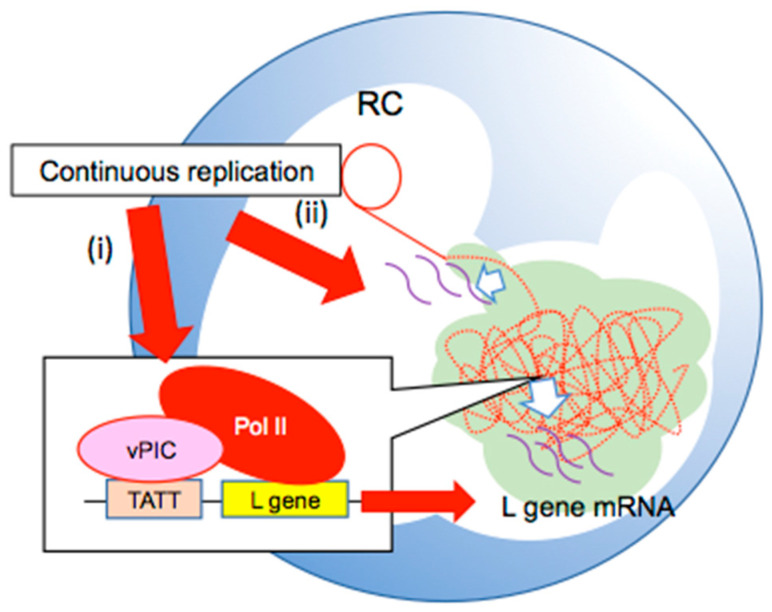
The interaction between continuous replication and vPIC-dependent or -independent L gene transcription in RCs. (i) Continuous replication is required by vPIC-dependent L gene transcription. vPIC, including viral TBP-like protein, interacts with the TATT motif on the L promoter region of viral DNA stored in the BMRF1-core. (ii) Some sets of L genes are transcribed independently of vPIC. Viral DNA replication stimulates such gene transcription.

RCs are also transcription sites. The major breakthrough regarding EBV transcription during the lytic phase was the discovery of the viral pre-initiation complex (vPIC). In the EBV L promoter region, a TATT motif is present instead of the TATA box that is normally found in eukaryotic promoters [84,85,86]. BcRF1 has been identified as a TATA-binding protein (TBP)-like protein that interacts with the TATT motif on the L promoter region and is essential for L gene transcription [46]. Six viral proteins (BDLF3.5, BDLF4, BVLF1, BGLF3, BFRF2, and BcRF1) compose the vPIC, all of which are necessary for L gene transcription [45]. This transcriptional system via vPIC is conserved among beta- and gamma- but not alpha-herpesviruses. Some set of vPIC components are stabilized by host factors such as CDK2, an S-phase-like CDK that is activated during the lytic phase through the phosphorylation of BDLF4 [87]. Herpesviral TBP-like proteins, such as BcRF1, can directly bind to host RNA polymerase II (Pol II) via the N-terminal domain, although cellular TBP does not normally bind to Pol II [88]. Confocal microscopy has revealed that Pol II accumulates inside the BMRF1-core at the late stage of the lytic cycle, while at the early stage Pol II is located outside the BMRF1-core [47]. BcRF1, which is considered to bind to Pol II, and transcribed mRNAs of L genes are localised inside the BMRF1-core, whereas the mRNAs of E genes are located outside the BMRF1-core [47]. vPIC-mediated L gene transcription requires continuous viral lytic replication [89] and seems to template mature and stored newly synthesized viral DNA in the BMRF1-core (Figure 4) [90].

In contrast, although vPIC is essential for almost all L genes, some sets of L genes, such as BCRF1 (vIL10) and BPLF1, do not require vPIC [91,92]. The “leaky” transcription of these genes is activated in a DNA replication-dependent manner and is caused by superimposition of both early and late transcription at the same promoter [90,91]. BGLF4 kinase activity elevates both vPIC-dependent and -independent L-gene transcription [92,93]. Therefore, continuous lytic DNA synthesis at RCs is required for both “true” and “leaky” L gene transcription (Figure 4) [55].

Additionally, the EBV-coded transcriptional factors BZLF1, Rta, and BMRF1 act as mediators of L gene transcription. Rta interacts with host factors such as TSG101 at RCs and upregulates L gene transcription [94]. The region encoding the transcription activity of the L gene in BZLF1 is involved in RC formation [95]. A subset of L genes classified as “true late” are partially transcribed in response to cellular transcriptional factors, such as AP-1 proteins with alanine-to-serine mutations, independently of viral DNA replication [96,97]. The interaction of BMRF1 with the SWI/SNF chromatin modifier subset BRG1 also upregulates L gene transcription [98]. Taken together, the roles of RCs in L gene transcription are: (1) acting as sites of vPIC-mediated transcription for storing viral DNA in the BMRF1-core, (2) allowing continuous replication, which leads to L gene transcription in vPIC-dependent and -independent manners, and (3) recruiting most EBV-coded transcription mediators and their interaction partners. RC formation may provide advantages for the interactions among viral transcriptional factors, cellular factors, and viral DNA.

## 7. The Indirect Maintenance of RCs

Viral and cellular factors, even those that do not localize to RCs, partly assist the formation and maintenance of RCs. One of the heat shock proteins (Hsps), Hsp90, is a well-conserved molecular chaperone that mediates molecule maturation, stabilization, and intracellular trafficking [99]. Since BALF5 does not possess nuclear localization signals (NLSs), Hsp90 beta, which is localized in the cytoplasm, mediates the interaction between BALF5 and BMRF1, facilitating the nuclear translocation of BALF5 [100]. 

APOBEC3B (A3B) is a member of the APOBEC family of ssDNA cytosine deaminases, which participate in antiviral innate immunity, and seems to be antagonistic to EBV viral gene stability, as A3B catalytic activity mediates deamination of cytosine to uracil in ssDNA. BORF2, an EBV ribonucleotide reductase, interacts with A3B and is transported to the cytoplasm from the nucleus, although deletion of BORF2 leads to the localization of A3B to RCs and reduces viral production [101]. Such protection systems by viral ribonucleotide reductases are highly conserved among alpha- and gamma-herpesviruses [102]. This shows that viral DNA stored in the RC is well protected from the cellular antiviral innate immune response.

## 8. Conclusions and Future Directions

Once EBV reactivation occurs, RC formation is observed. The four reasons for RC formation are: (1) RCs enable the spatial separation of newly synthesized viral DNA from the cellular chromosome for viral DNA protection and maturation; (2) EBV-coded proteins and their interaction partners are recruited to RCs, which enhances interactions among viral proteins, cellular proteins, and viral DNA; (3) RC formation facilitates continuous replication, leading to L gene transcription; and (4) DNA storage and maturation leads to efficient progeny viral production. These aspects benefit EBV, since viral genomes can be amplified rapidly and immediately, in addition to maintaining stability by interacting with host factors, such as DNA repair factors, and evasion of some DDR factors or A3B. Most viral genes that encode structural proteins are classified as L genes [9]. Both efficient L gene transcription and genome stability are essential for virion production. RCs play an important role in almost all phases of lytic replication.

Recently, the period from primary infection to the establishment of latency, termed the pre-latent phase, has attracted great interest because early events are directly linked to EBV-driven transformation [103,104]. During the pre-latent phase, latent genes, as well as some sets of lytic genes, are expressed temporarily [103,105]. DDR is also temporarily induced and attenuated until latency is established [106]. However, unlike lytic replication, cellular DNA synthesis and cell division occur several days after primary infection, progeny viruses are not produced, and BZLF1 is not necessary for infection during the pre-latent phase [107,108]. Thus, because cellular aspects during the pre-latent phase differ from those of the lytic phase, little is known about how viral DNAs are synthesized, including whether RCs are formed, although viral DNA is abundantly amplified during the pre-latent phase. Moreover, recently it has been suggested that cells during the “abortive lytic phase” exist [109]. The “abortive lytic phase” is, so to speak, an “incomplete lytic cycle”, wherein several early lytic genes are expressed without progeny viral production like the pre-latent phase [108,110]. Studying these “incomplete lytic phases”, including whether RCs are assembled or not, might unveil the novel significance of RC formation.

## Figures and Tables

**Table 1:** Viral proteins accumulate to RCs. “Microscopic analysis” means that the localization to RCs of the viral protein was improved by IFA. “N/A” indicates that the localization to RCs of the viral protein was not improved by IFA but that it was estimated to accumulate in RCs by co-immunoprecipitation analysis with viral proteins recruited to RCs or other functional assays.

Viral Protein	Functions	Roles in RCs	Microscopic Analysis	Refs.
EBNA1	Bridge between chromosome and viral episome	Scaffold for newly synthesized DNA	Yes	[19]
BZLF1	Lytic cycle switch	Activates and modulates DDR L gene transcription	Yes	[19]
Rta	Transcriptional activator	L gene transcription	Yes	[19]
BALF2	ssDNA binding protein	Consists of viral replication forks and synthesizes viral DNA	Yes	[19]
BALF5	DNA polymerase	Consists of viral replication forks and synthesizes viral DNA	Yes	[19]
BMRF1	dsDNA binding protein polymerase processsivity factor	Consists of viral replication forks and synthesizes viral DNA Binds to dsDNA and composes the BMRF1-core Mediates DNA repair L gene transcription	Yes	[19]
BBLF2/3	Helicase–primase complex	Consists of viral replication forks and synthesizes viral DNA	Yes	[19]
BBLF4	Helicase–primase complex	Consists of viral replication forks and synthesizes viral DNA	Yes	[19]
BSLF1	Helicase–primase complex	Consists of viral replication forks and synthesizes viral DNA	Yes	[19]
BKRF3	Uracil DNA glycosylase	Assists in the synthesis of viral DNA	Yes	[38]
BGLF4	Protein kinase	Conducts chromosomal condensationActivates DDR	Yes	[39]
BPLF1	Deubiquitinase	Mediates DNA repair	Yes	[40]
BGLF5	Alkaline nuclease	Viral mRNA export	Yes	[41]
BMLF1	Transcriptional activator	Viral mRNA export	Yes	[41]
BFRF3	Small capsid protein	Consists of viral capsids	Yes	[42]
BVRF1	Minor capsid protein/Capsid packaging protein	Packages viral DNA into capsids	Yes	[42]
BGLF1	Minor capsid protein/Capsid packaging protein	Packages viral DNA into capsids	Yes	[42]
BFLF1	Capsid packaging protein	Packages viral DNA into capsids	Yes	[42]
BDRF1	Minor capsid protein/Capsid packaging protein	Packages viral DNA into capsids	N/A	[43]
BVRF2	Capsid protease	Consists of viral capsids	Yes	[42]
BDLF1	Capsid Triplex 2	Consists of viral capsids	Yes	[42]
BORF1	Capsid Triplex 1	Consists of viral capsids	Yes	[42]
BBRF1	Capsid portal protein	Consists of viral capsids	Yes	[42]
BdRF1	Capsid scaffold protein	Consists of viral capsids and is cleaved	N/A	[44]
BcLF1	Major capsid protein	Consists of viral capsids	N/A	[44]
BDLF3.5	vPIC component	L gene transcription	N/A	[45]
BDLF4	vPIC component	L gene transcription	N/A	[45]
BVLF1	vPIC component	L gene transcription	N/A	[45]
BGLF3	vPIC component	L gene transcription	N/A	[45]
BFRF2	vPIC component	L gene transcription	N/A	[45]
BcRF1	vPIC component	L gene transcription	Yes	[46,47]
